# Cardiac autonomic function in adults born preterm with very low birth weight in mid‐adulthood—A two‐country birth cohort study

**DOI:** 10.14814/phy2.70641

**Published:** 2025-10-29

**Authors:** Laura Jussinniemi, Zareen Tasnim, Mikko Tulppo, Tora Sund Morken, Kari Anne I. Evensen, Eero Kajantie

**Affiliations:** ^1^ Clinical Medicine Research Unit University of Oulu Oulu Finland; ^2^ Welfare Epidemiology and Monitoring Unit Finnish Institute for Health and Welfare Helsinki Finland; ^3^ Epidemiology and Biomedical Data Science University of Oulu Oulu Finland; ^4^ Research Unit of Biomedicine and Internal Medicine University of Oulu Oulu Finland; ^5^ Medical Research Center Oulu Oulu University Hospital and University of Oulu Oulu Finland; ^6^ Department of Neuromedicine and Movement Science Norwegian University of Science and Technology (NTNU) Trondheim Norway; ^7^ Department of Ophthalmology, St. Olavs Hospital Trondheim University Hospital Trondheim Norway; ^8^ Department of Clinical and Molecular Medicine NTNU Trondheim Norway; ^9^ Department of Rehabilitation Science and Health Technology Oslo Metropolitan University Oslo Norway; ^10^ Children's Clinic, St. Olavs Hospital Trondheim University Hospital Trondheim Norway

**Keywords:** adulthood, cardiac autonomic function, heart rate variability, prematurity, very low birth weight

## Abstract

Cardiac autonomic functioning is altered in children and young adults born preterm with very low birth weight (VLBW; <1500 g). Whether these alterations persist into mid‐adulthood remains unknown. We studied heart rate variability (HRV) in two birth cohorts, HeSVA (Finland) and NTNU LBW Life (Norway), with harmonized methods. HRV was assessed in 107 adults born preterm with VLBW and 142 controls born term with normal birth weight at a mean age of 36 (SD 3.3) years. We hypothesized that adults born preterm with VLBW have lower parasympathetic activity and higher blood pressure (BP), partly mediated by lower parasympathetic activity. Participants born preterm with VLBW had higher heart rate and BP than controls. In sex‐stratified analyses, mean differences in high‐frequency (HF) power were −43.3% (95% CI −63.9%, −11.3%) in women and −36.9% (−65.0%, 15.0%) in men. For root mean square of successive differences, differences were −18.2% (−35.6%, 4.1%) in women and 18.5% (−10.4%, 58.4%) in men. Low‐frequency (LF) power differed by −23.7% (−46.2%, 10.5%) in women and 35.0% (−16.5%, 120.3%) in men. LF/HF ratio was 36.3% (4.1%, 76.8%) higher in women and −13.9% (−34.3%, 12.7%) in men. Among women, elevated BP was partly mediated by HRV. Findings suggest altered autonomic regulation in adults born preterm with VLBW, especially women, potentially contributing to higher BP.

## 1 INTRODUCTION

### 1.1 Background

Worldwide approximately 10% of infants are born preterm, <37 completed weeks of gestation (Lawn et al., 2023), and 1% with very low birth weight (VLBW; <1500 g) (March of Dimes Peristats, 2025). Previous studies have demonstrated that prematurity is associated with lifelong alterations and abnormalities in cardiovascular function (Johansson et al., 2007; Patural et al., 2004; Yiallourou et al., 2013), predisposing individuals born preterm to a higher risk of cardiovascular disease later in life (Barker, 1995; Chehade et al., 2018; Karvonen et al., 2019; Kumaran et al., 2017).

Impaired cardiac autonomic function, characterized by reduced vagal activity and increased sympathetic activity, represents a significant risk factor for cardiovascular diseases (Thayer et al., 2010; Thayer & Lane, 2007; Tsuji et al., 1996). Much of the development of the autonomic nervous system such as vagus nerve myelination, baroreflex sensitivity, and heart rate variability (HRV) takes place during the third trimester of pregnancy and is disrupted by preterm birth (Andriessen et al., 2005). This could impact cardiac autonomic regulation later in life (Van Leeuwen et al., 1999; Yiallourou et al., 2013), which could serve as a mechanism connecting preterm birth to increased blood pressure and increased risk for cardiovascular disease in adulthood (Karvonen et al., 2019).

HRV measurement is a widely used method to assess cardiac autonomic function (Thayer & Lane, 2007). There is emerging evidence that preterm birth is a risk factor for impaired cardiac autonomic control later in life (Karvonen et al., 2019; Mathewson et al., 2015; Rakow et al., 2013; Yiallourou et al., 2013). Elevated blood pressure (BP) has also been consistently observed in individuals born preterm (Hovi et al., 2016; Parkinson et al., 2013). For example, a recent individual participant meta‐analysis showed that in adults born preterm with VLBW, systolic BP was on average 1.8 mmHg higher in men (95% CI 0.1–3.5) and 4.7 mmHg higher in women (95% CI 3.2–6.3) compared with term‐born peers (Hovi et al., 2016); however, the mechanisms are poorly known. One proposed mechanism linking preterm birth to elevated BP is impaired autonomic regulation, where reduced parasympathetic and/or increased, sympathetic activity may contribute to long‐term cardiovascular risk (Thayer et al., 2010).

Sex differences may further influence the association between preterm birth, autonomic function, and blood pressure. In the general population, women typically show higher parasympathetic activity and lower sympathetic activity than men, as reflected in HRV indices (Koenig & Thayer, 2016; Shaffer & Ginsberg, 2017; Thayer & Lane, 2007). However, studies specifically examining sex‐related variation in HRV among adults born preterm remain scarce. Initial findings suggest possible sex‐specific patterns, such as lower HRV in preterm‐born men but no clear differences in women compared with term‐born peers; overall, the results remain inconclusive (Björkman et al., 2023; Karvonen et al., 2019).

The earliest generations of preterm born infants with VLBW who received neonatal intensive care are now reaching middle age. However, there are limited published data on cardiac autonomic function in individuals born preterm with VLBW during mid‐adulthood.

### 1.2 Objectives

We assessed cardiac autonomic function by HRV in adults born preterm (<37 completed weeks of gestation) with VLBW (<1500 g) at a mean age of 36 (SD 3.3) years and compared them with term‐born peers. Our primary hypothesis was that adults born preterm with VLBW adults have reduced cardiac parasympathetic activity compared with those born at term. In addition, we hypothesized that adults born preterm with VLBW would have higher blood pressure compared with their term‐born counterparts with normal birth weight, and that this potential difference would be partly mediated by lower parasympathetic activity. Because of previous evidence suggesting that the differences in blood pressure between born preterm with VLBW and comparison adults are larger among women (Bates et al., 2020; Hovi et al., 2016), we present the data separately for women and men.

## 2 MATERIALS AND METHODS

### 2.1 Study design

The data from two longitudinal birth cohorts, the Helsinki Study of Very Low Birth Weight Adults (HeSVA, Helsinki, Finland) and the Norwegian University of Science and Technology Low Birth Weight in a Lifetime Perspective Study (NTNU LBW Life, Trondheim, Norway) were collected with harmonized methods. The follow‐up clinical visits were conducted during 2019–2021 with comprehensive health assessments including HRV measurements.

The original HeSVA cohort comprises 335 infants born preterm with VLBW born in 1978–1985 and discharged alive from the neonatal intensive care unit (NICU) of Helsinki University Central Hospital. The cohort has undergone comprehensive clinical assessments in young adulthood at mean ages of 22 and 25 years. A control group was selected at the 22‐year visit, group‐matched for age, sex and birth hospital (Hovi et al., 2007; Jussinniemi et al., 2023; Kajantie et al., 2008).

The original NTNU LBW Life cohort included 88 infants born preterm with VLBW born in 1986–1988 who were admitted and discharged alive from the NICU at St. Olavs Hospital, Trondheim, Norway. The cohort has undergone detailed clinical examinations at ages 1, 5, 14, 18, 20, 23, and 26. The control participants were born not small for gestational age to women from the Trondheim region and were enrolled during pregnancy before gestational week 20 of their mothers' second or third pregnancy in a multicenter study on causes and consequences of intrauterine growth restrictions (Bakketeig et al., 1993; Jussinniemi et al., 2023).

### 2.2 Participants

**FIGURE 1:**
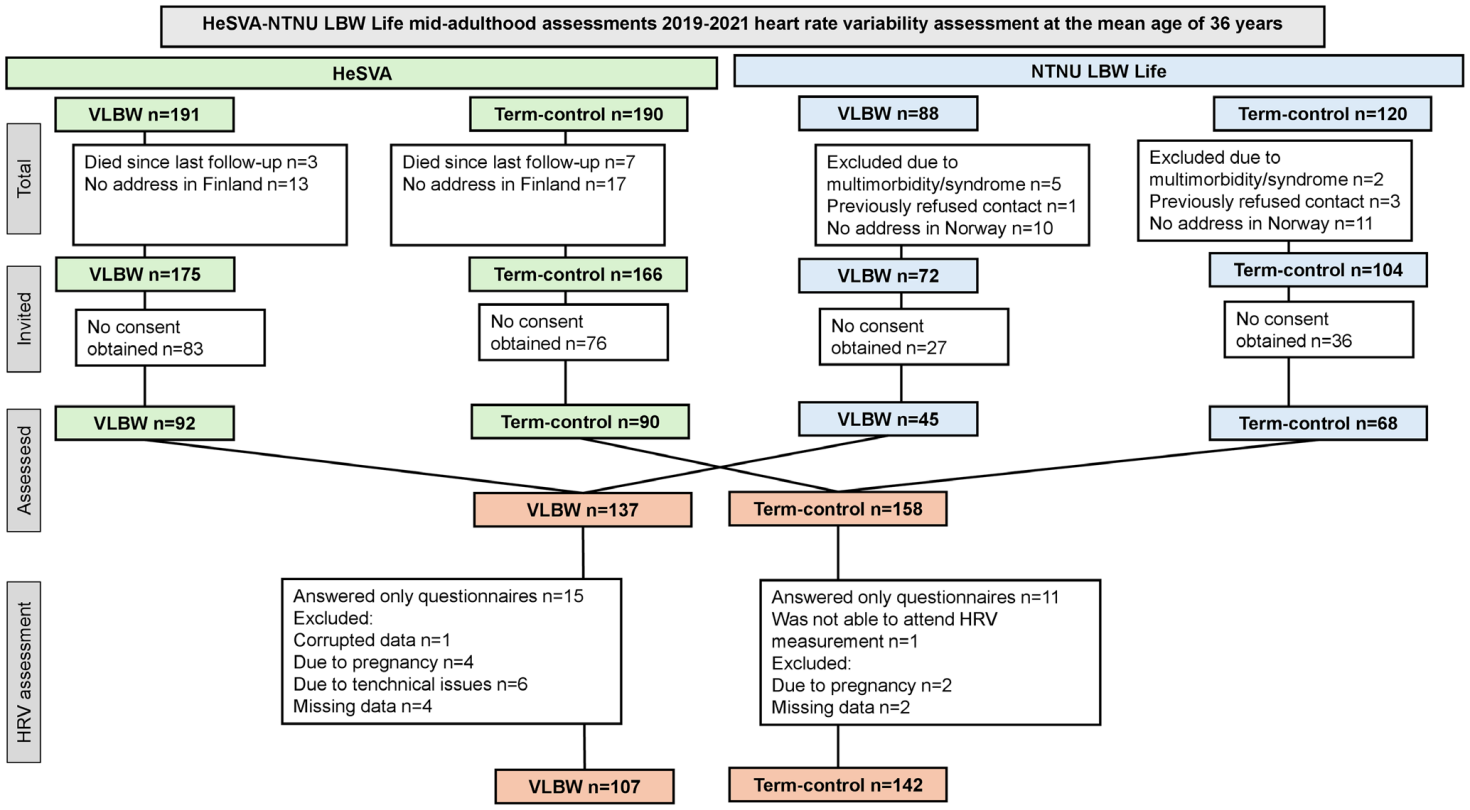
Flow of the HeSVA‐NTNU LBW Life heart rate variability assessment participants. HeSVA, Helsinki Study of Very Low Birth Weight; HRV, heart rate variability; NTNU LBW Life, NTNU Low Birth Weight in a Lifetime Perspective Study.

Figure 1 illustrates the flow of study participants. A total of 175 VLBW adults born preterm from HeSVA and 72 from NTNU LBW Life were invited for mid‐adulthood assessment at the mean age of 36 (SD 3.3) years. Staff at both study sites received similar training and consistency of performance was confirmed in audits. Of those invited, 137 participated (55.5% women), of whom 15 answered questionnaires only and did not attend the clinical visits. Four participants were excluded from the HRV analysis due to pregnancy and 11 due to technical issues, missing or corrupted data. In the HRV analysis, we thus included 107 participants who were born preterm with VLBW (55.1% women).

In the control group, participants born term and with normal birth weight, 166 from HeSVA and 104 from NTNU LBW Life were invited. Of them, 158 (58.5% women) participated. Eleven control participants answered only questionnaires and did not attend the clinical visits. Two participants were excluded from the analysis due to pregnancy and three due to technical issues, missing or corrupted data. In the HRV analyses we included 142 control participants (57.0% women).

### 2.3 Measures

HRV recordings were conducted in a standard health examination room as part of a broader health assessment that included questionnaires, physiological measurements, eye examination, spirometry, and other clinical tests. The participants were asked to refrain from smoking and using snuff 3 h before the clinical visit. In addition, the participants were asked to avoid use of alcohol and not engage in vigorous physical exercise the previous day.

HRV was measured by the participants wearing a Polar heart rate monitor belt (Polar Electro Oy, Kempele, Finland) in a resting position at spontaneous breathing rate. Five minutes of resting periods were recorded by a study nurse and the HRV, the changes in the time intervals between consecutive heartbeats (R‐R intervals), recordings were pooled across the cohorts. To evaluate cardiac autonomic function, the following time and frequency domains of HRV were obtained: the mean heart rate (HR), root mean square of successive differences (rMSSD), low frequency (LF) power (LFP, 0.04–0.15 Hz), high frequency (HF) power (HFP,0.15–0.40 Hz), and the ratio between LF and HF (LF/HF). Based on prior literature and biological relevance (*European Heart Journal*, 1996; Shaffer & Ginsberg, 2017), rMSSD and HFP were prespecified as the primary outcomes of interest, reflecting parasympathetic cardiac regulation.

In addition, during the clinical visits, office blood pressure was measured from the right arm in a sitting position after a minimum of 5 min rest by an automated blood pressure monitor three times with 1‐min intervals. The mean of the three measurements was calculated for the analysis.

Information on the study participants' and maternal background characteristics (Table 1) was obtained from medical records. Data on education, daily smoking, diseases, and medication usage were collected through a self‐report questionnaire. Physical activity was assessed by accelerometry (Benum et al., 2025).

**TABLE 1:** Background characteristics of HeSVA‐NTNU LBW life participants.

	VLBW	Term (reference)	
Number of participants	107	142	
HeSVA	78	83	
Women	44	47	
Men	34	36	
NTNU	29	59	
Women	15	34	
Men	14	25	

	Mean (SD) or *n* (%)	Mean (SD) or *n* (%)	*p* Value
Maternal characteristics			
Age of the mother (years)	29.8 (4.8)	30.2 (5.0)	0.58
Smoking during pregnancy^a^	13 (23.6%)	11 (17.2%)	0.38
Parental education			
Basic or less	17 (16.3%)	15 (11.5%)	0.13
Upper secondary	20 (19.2%)	28 (21.4%)	
Lower‐lever tertiary	38 (36.5%)	35 (26.7%)	
Upper‐level tertiary	29 (27.9%)	53 (40.5%)	
Study participant background			
Sex, women	59 (55.1%)	81 (57.0%)	0.77
Gestational age (weeks)	29.3 (2.4)	40.0 (1.1)	<0.001
Birth weight (g)	1158 (220)	3646 (465)	<0.001
Birth weight SD score Finnish reference^b^	−1.30 (1.61)	0.13 (1.00)	<0.001
Birth weight SD score Norwegian reference^b^	−1.06 (1.12)	0.04 (0.98)	<0.001
Cerebral palsy	7 (6.5%)	1 (0.7%)	0.02
Ventilator treatment (days)	8.59 (14.88)	—	
Supplemental oxygen (days)	19.71 (24.94)	—	
Age at discharge from hospital (days)	67.74 (25.5)	—	
Bronchopulmonary dysplasia			
Defined as supplemental oxygen >28 days	30 (29.4%)	—	
Defined as supplemental oxygen >36 weeks	8 (7.8%)	—	
Diagnosed by a clinician	19 (24.1%)	—	
VLBW and SGA^c^	43 (40.2%)	—	
Study participant current characteristics			
Age (years)	36.5 (3.1)	35.8 (3.3)	0.08
Educational attainment			
Lower (ISCED levels 1–2)	4 (3.7%)	2 (1.4%)	0.02
Intermediate (ISCED levels 3–5)	51 (47.7%)	47 (33.1%)	
Lower tertiary or higher (ISCED levels 6–8)	52 (48.6%)	93 (65.5%)	
Height (cm)	168.3 (9.8)	173.8 (9.6)	<0.001
Women	163.0 (7.9)	167.5 (6.1)	<0.001
Men	174.8 (7.9)	182.2 (6.4)	<0.001
BMI kg/m^2^	26.2 (6.2)	25.6 (4.4)	0.45
Women	26.4 (7.1)	25.8 (5.0)	0.27
Men	25.8 (4.9)	25.5 (3.5)	0.33
Smoking daily (yes/no)	12 (11.2%)	15 (10.6%)	0.87
Physical activity, device measured (MetMin/day)			
Inactive (sitting)			
women	517 (127)	527 (127)	0.70
men	653 (147)	592 (153)	0.07
Light (standing/slow walking)			
Women	744 (204)	774 (160)	0.41
Men	572 (183)	603 (204)	0.46
Moderate‐to‐vigorous activity			
Women	172 (96)	193 (114)	0.34
Men	129 (66)	181 (115)	0.01
Hormonal contraception (women)	20 (33.3%)	35 (43.2%)	0.24
Diseases and medications reported during clinical visit			
Type II diabetes mellitus^d^			
Women	1 (1.7%)	1 (1.2%)	0.672
Men	3 (6.4%)	0 (0%)	0.079
High blood pressure^d^			
Women	6 (10.2%)	0 (0%)	0.005
Men	5 (10.6%)	3 (4.9%)	0.29
Heart disease^d^			
Women	1 (1.7%)	1 (1.2%)	1.00
Men	1 (2.1%)	0 (0%)	0.44
B‐blockers (ATC = C07A)			
Women	2 (3.3%)	1 (1.2%)	0.58
Men	0 (0%)	3 (4.9%)	0.26
Agents acting on the renin‐angiotensin system (ATC = C09)			
Women	1 (1.7%)	0 (0%)	0.43
Men	4 (8.5%)	3 (4.9%)	0.47
Lipid modifying agents (ATC = C10)			
Women	—	—	
Men	3 (6.4%)	1 (1.6%)	0.32
Psycholeptics (ATC = N05)			
Women	9 (15%)	1 (2.1%)	0.002
Men	1 (2.1%)	2 (3.3%)	1.00
Psychostimulants (ATC = N06B)			
Women	2 (3.3%)	0 (0%)	0.18
Men	1 (2.1%)	1 (1.6%)	1.00
Inhaled adrenergic bronchodilators (ATC = R03AC)			
Women	11 (5.9%)	3 (3.7%)	0.004
Men	4 (8.3%)	6 (9.8%)	0.79
Calcium channel blockers	—	—	
Medication for DM II (ATC = A10B, no insulins)			
Women	2 (3.4%)	0 (0%)	0.18
Men	2 (4.2%)	0 (0%)	0.19
Use of any asthma medication for obstructive airways disease (R03AC, R03AK, R03BA, and R03DC)	18 (16.8%)	11 (7.7%)	0.03
Women	13 (22.0%)	5 (6.2%)	0.006
Men	5 (10.4%)	6 (9.8%)	1.0

*Note*: Comparisons between adults born preterm with VLBW and term‐controls done by x^2^ and *t*‐tests.

Abbreviations: ATC, The Anatomical Therapeutic Chemical code; BMI, body mass index; HeSVA, Helsinki Study of Very Low Birth Weight Adults; ISCED, International Standard Classification of Education; NTNU, NTNU Low Birth Weight in a Lifetime Perspective Study; SD, standard deviation; SGA, small for gestational age; VLBW, very low birth weight.

^a^ Data available only from HeSVA participants.

^b^ In HeSVA based on Pihkala standards (Pihkala et al., 1989) and in NTNU LBW Life on Norwegian growth curves.

^c^ Defined as: HeSVA, birth weight <2 SD and NTNU LBW Life, birth weight <10th percentile according to Norwegian growth curves (Skjaerven et al., 2000).

^d^ History of disease diagnosed by a clinician.

### 2.4 Sample size

A priori power calculation was based on a total sample estimated to attend this follow‐up assessment, 170 preterm‐born VLBW participants and 200 controls. With a statistical power of 80% and an alpha level of 0.05, the detectable difference in a continuous outcome between groups was 0.29 SD score and with 90% power and an alpha of 0.01 the detectable difference was 0.40 SD. Before the analysis, with the actual number of 137 participants born preterm with VLBW and 158 control participants, the corresponding numbers were 0.33 and 0.45 SD (Jussinniemi et al., 2023). In sex‐specific analyses comparing 48 men born preterm with VLBW with 61 control men (less represented sex), the corresponding numbers were 0.55 and 0.76 SD and comparing 89 women born preterm with VLBW with 97 control women they were 0.42 and 0.57 SD.

### 2.5 Statistical analyses

The data were analyzed with IBM SPSS statistics, version 29.0.0.0 (241).

The data were processed by using Hearts 1.2, a University of Oulu (Finland) in‐house software script. All R‐R interval data were visually inspected beat‐by‐beat to detect the artifacts and ectopic beats to be replaced with the local average. If 10 or more consecutive beats, artifacts or disturbances were found, they were removed. To be included in the analysis each participant's HRV data must contain R‐R series with at least 4 min with a minimum of 90% accepted R‐R intervals. We then calculated the mean HR, rMSSD, LFP, HFP, and LF/HF by the Hearts software and expectedly found skewed distributions, which is why we transformed these measurements into natural logarithm values prior to the analysis. These were transformed back and are reported as geometric means and percentage differences.

The comparisons of the baseline, background, diagnosed diseases, and medication usage characteristics between participants born preterm with VLBW and term‐born controls were done by x^2^ and *t*‐tests.

We used linear regression to detect differences between preterm birth with VLBW and control groups. To evaluate whether the associations differed by sex between preterm birth with VLBW and HRV, we did interaction analysis by using the term group*sex for rMSSD, LFP, HFP, and LF/HF. We found that HFP differed significantly between sexes, women having significantly lower values. All linear regressions were adjusted for age, sex (if not stratified), and cohort (Model 1). In addition, we conducted additional adjustment models. Model 2 including highest parental educational attainment and Model 3 including hormonal contraception in addition to model 1 variables. Model 3 analysis was done only in women.

Since HRV may be affected by several diseases, medications and health conditions (Stapelberg et al., 2012), sensitivity analyses were performed with different group selections; sensitivity analysis 1 excluded the participants with cerebral palsy (CP) or type II diabetes (DMII) or heart diseases diagnosed by a clinician and self‐reported usage of the following medications: agents acting on the renin‐angiotensin system (ATC = C09), adrenergic bronchodilators (ATC = R03AC), DMII (ATC = A10B), B‐blockers (ATC = C07A), lipid‐modifying agents (ATC = C10), psycholeptics (ATC = N05), or psychostimulants (ATC = N06B). Sensitivity analysis 2 was similar to sensitivity analysis 1, but those who reported usage of psycholeptics or psychostimulants were included in the analysis. Sensitivity analysis 3 excluded the participants with CP, heart disease and those who reported usage of agents acting on the renin‐angiotensin system or B‐blockers, and sensitivity analysis 4 excluded only the participants with CP.

**FIGURE 2:**
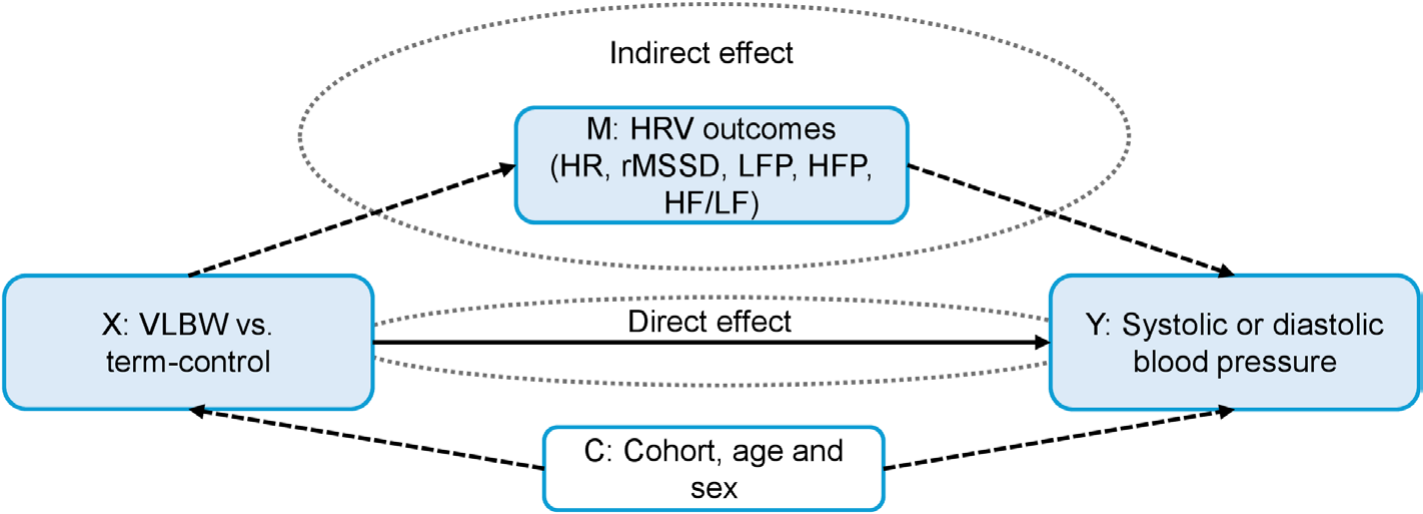
Relationship between adults born preterm birth with VLBW (X), HRV metrics (M), and systolic and diastolic blood pressure (Y) with confounders (C). HFP, high frequency power; LF/HF, ratio between low and high frequency power; HR, mean heart rate; HRV, heart rate variability; LFP, low frequency power; rMSSD, root mean square of successive differences; VLBW, very low birth weight.

To assess the direct, indirect and total effect of preterm birth with VLBW on systolic and diastolic blood pressure with HRV outcomes as mediators, we performed mediation analyses using the PROCESS macro for IBM SPSS and calculated 95% confidence intervals based on 5000 bootstrap samples (Hayes & Rockwood, 2017). In the model (Figure 2), HRV outcomes (HR, rMSSD, LFP, HFP, and LF/HF) were separately set as the mediator variables (M), group (preterm birth with VLBW vs. term control) as the predictor variable (X), and systolic and diastolic blood pressure separately as the outcome variables (Y). Mediation analyses were adjusted for age, sex, and cohort (C).

In addition, we performed nonparticipant analyses between the participants who were included in the HRV analyses (participants) and those who attended the mid‐adulthood study but were not included in the HRV analyses (nonparticipants).

All results are reported as mean differences with corresponding 95% confidence intervals (95% CI), unless otherwise specified. Statistical significance was set at *p* < 0.05.

## 3 RESULTS

### 3.1 Background characteristics of the clinical visits

Background characteristics are shown in Table 1. Participants born preterm with VLBW had lower educational attainment (*p* = 0.022) and men born preterm with VLBW undertook less physical activity in the moderate to vigorous category (*p* = 0.011) compared with controls. In addition, women born preterm with VLBW more often reported high blood pressure diagnosed by a clinician (*p* = 0.005) and participants born preterm with VLBW more often reported usage of psycholeptics and asthma medications (Table 1).

### 3.2 Heart rate variability in adults born preterm with VLBW

**FIGURE 3:**
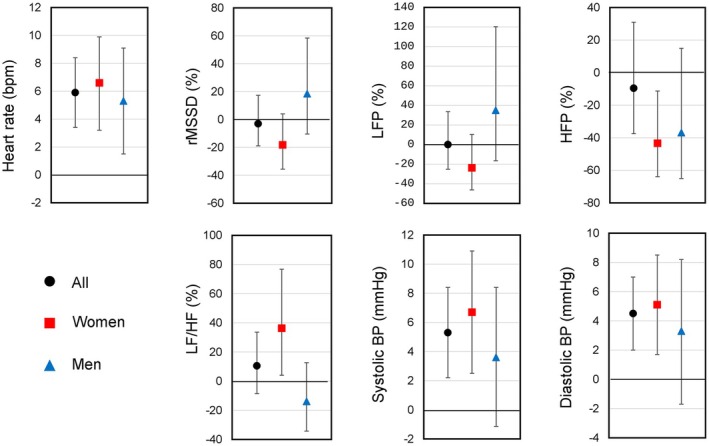
Mean differences (95% CI) in heart rate variability and blood pressure outcomes in adults born preterm with VLBW compared with term‐born controls adjusted for cohort, age, and sex (if not stratified). rMSSD, LFP, HFP, and LF/HF presented from back transformed values as percentage differences. CI, confidence interval; HFP, high frequency power; HF/LF, ratio between high and low frequency power; HR, mean heart rate; HRV, heart rate variability; LFP, low frequency power; rMSSD, root mean square of successive differences; VLBW, very low birth weight.

The HRV and blood pressure geometric means and percentage differences are described in Table 2 and Figure 3. Adjusted for age and cohort, women and men born preterm with VLBW had higher resting heart rates compared with term‐born controls. In addition, women born preterm with VLBW had lower HFP (−43.3%; −63.9% to −11.3%) indicating weaker vagal variability in respiratory frequency and higher LF/HF (36.3%; 4.1% to 76.8%) indicating sympathetic dominance in relation to parasympathetic, compared with control women. There were no differences in HRV between adults born preterm with VLBW and control men except in resting heart rate (5.3 bpm; 1.5–9.1) shown in Table 2 and Figure 3.

**TABLE 2:** HRV and blood pressure outcomes in adults born preterm with VLBW and term control participants and their mean differences (95% CI).

	VLBW/term	VLBW	Term (reference)	Mean difference (95% CI)^b^
	*n*	Mean (SD)	Mean (SD)	
Resting HR (bpm)				
Pooled	107/142	73.7 (10.5)	67.5 (10.0)	5.9 (3.4 to 8.4)
Women	59/81	75.8 (8.7)	69.4 (10.4)	6.6 (3.2 to 9.9)
Men	48/61	71.1 (11.9)	65.0 (8.9)	5.3 (1.5 to 9.1)
rMSSD (ms)^a^				
Pooled	107/142	44.7 (2.3)	47.5 (2.0)	−3.0% (−18.9% to 17.4%)
Women	59/81	37.0 (1.9)	46.1 (2.1)	−18.2% (−35.6% to 4.1%)
Men	48/61	55.7 (2.6)	49.4 (1.9)	18.5% (−10.4% to 58.4%)
LFP (ms^2^)^a^				
Pooled	107/142	1188.0 (3.7)	1224.1 (2.9)	0.10% (−25.2% to 33.6%)
Women	59/81	837.1 (2.7)	1085.7 (2.9)	−23.7% (−46.2% to 10.5%)
Men	48/61	1826.2 (4.6)	1436.6 (2.8)	35.0% (−16.5% to 120.3%)
HFP (ms^2^)^a^				
Pooled	107/142	780.6 (5.3)	906.9 (3.6)	−9.5% (−37.5% to 31.0%)
Women	59/81	533.8 (3.6)	934.5 (3.7)	−43.4% (−63.9% to −11.3%)
Men	48/61	1249.0 (7.2)	880.1 (3.5)	−36.9% (−65.0% to 15.0%)
LF/HF^a^				
Pooled	107/142	1.5 (2.1)	1.3 (2.1)	10.5% (−8.6% to 33.6%)
Women	59/81	1.6 (2.0)	1.2 (2.3)	36.3% (4.1% to 76.8%)
Men	48/61	1.5 (2.2)	1.6 (1.9)	−13.9% (−34.3% to 12.7%)
Systolic blood pressure (mmHg)				
Pooled	105/141	117.4 (14.8)	111.5 (12.2)	5.3 (2.2 to 8.4)
Women	57/80	113.3 (14.5)	106.00 (10.1)	6.7 (2.5 to 10.9)
Men	48/61	122.3 (13.9)	118.8 (10.8)	3.6 (−1.14 to 8.4)
Diastolic blood pressure (mmHg)				
Pooled	105/141	80.8 (11.2)	75.8 (8.3)	4.5 (2.0 to 7.0)
Women	57/80	79.8 (11.3)	74.4 (8.5)	5.1 (1.7 to 8.5)
Men	48/61	81.9 (11.2)	77.8 (7.7)	3.8 (0.15 to 7.4)

*Note*: Mean difference comparisons between adults born preterm with VLBW participants and term‐born controls made by linear regressions adjusted for age, cohort and sex (if not stratified).

Abbreviations: bpm, beats per minute; CI, confidence interval; HFP, high frequency power; HR, mean heart rate; LF/HF, ratio between low and high frequency power; LFP, low frequency power; ms, millisecond; ms^2^, square millisecond; rMSSD, root mean square of successive differences; SD, standard deviation; VLBW, very low birth weight.

^a^ Means (SD) for rMSSD, LFP, HFP and LF/HF are geometric means.

^b^ Mean differences for rMSSD, LFP, HFP and LF/HF have been calculated from log‐transformed values, back‐transformed and expressed as percentage difference with the same adjustment model.

Sensitivity analyses (1–4) excluding people with certain manifest diseases or medications (Table S1) and additional adjustment models accounting for parental education (Model 2) and participant use of hormonal contraception (Model 3; Table S2) showed little difference compared with adjustment Model 1.

### 3.3 HRV and its effect on blood pressure

The adults born preterm with VLBW group had higher systolic (5.3 mmHg; 2.2–8.4) and diastolic (4.5 mmHg; 2.0–7.0) BP compared with the control group. In sex‐stratified analyses, systolic BP was higher among women born preterm with VLBW compared with control women, but the corresponding difference was not seen among men (Table 2 and Figure 3).

Table 3 shows the results of the mediation analysis with direct, indirect, and total effects of the association between preterm birth with VLBW and BP, with HRV metrics as mediators. The indirect effect showed that among women, preterm birth with VLBW was associated with higher systolic blood pressure that was mediated through increased HR (3.0 mmHg; 1.1–5.9), HFP (2.1 mmHg; 0.4–4.4), and LF/HF (1.0 mmHg; 0.02–2.4) and significantly higher diastolic BP that was mediated through increased HR (2.9 mmHg; 1.1–5.7) and HFP (1.8 mmHg; 0.4–3.8). This suggests that HR, HFP, and LF/HF mediate the relationship between VLBW preterm birth and BP among women. Among men, the association between preterm birth with VLBW and higher diastolic BP was mediated through increased HR (1.7 mmHg; 0.2–3.8).

**TABLE 3:** Direct, indirect and total effect of preterm birth with very low birth weight on systolic and diastolic blood pressure with HRV metrics as mediators.

Outcome variable	HRV metrics as a mediator	Direct effect		Indirect effect		Total effect	
(Y)	(M)	(X)		(X)		(X)	
		Estimate (mmHg)	95% CI	Estimate (mmHg)	95% CI	Estimate (mmHg)	95% CI
Systolic blood pressure, mmHg	HR						
	Pooled	6.3	2.8 to 9.7	1.7	0.4 to 3.3	6.3	2.9 to 9.7
	Women	5.5	0.6 to 10.4	3.0	1.1 to 5.9	8.5	3.7 to 13.4
	Men	3.0	−1.9 to 8.0	0.6	−0.8 to 2.4	3.6	−1.1 to 8.4
	rMSSD						
	Pooled	6.2	2.8 to 9.5	0.1	−0.6 to 1.0	6.3	2.9 to 9.7
	Women	7.2	2.6 to 11.9	1.3	−0.1 to 3.3	8.5	3.7 to 13.4
	Men	3.8	−1.0 to 8.3	−0.2	−1.2 to 0.4	3.8	−1.0 to 8.6
	LFP						
	Pooled	6.3	2.9 to 9.7	0.01	−0.76 to 0.81	6.3	2.9 to 9.6
	Women	7.5	2.8 to 12.2	1.0	−0.3 to 3.0	8.5	3.7 to 13.4
	Men	3.9	−0.9 to 8.7	−0.3	−1.3 to 0.3	3.6	−1.1 to 8.4
	HFP						
	Pooled	6.1	2.7 to 9.4	0.2	−0.5 to1.2	6.3	2.9 to 9.7
	Women	6.4	1.7 to 11.1	2.1	0.4 to 4.4	8.5	3.7 to 13.4
	Men	3.8	−1.0 to 8.7	−0.2	−1.2 to 0.5	3.6	−1.1 to 8.3
	LF/HF						
	Pooled	6.1	2.7 to 9.7	0.2	−0.1 to 0.7	6.3	2.9 to 9.7
	Women	7.6	2.7 to 12.4	1.0	0.02 to 2.4	8.5	3.7 to 13.4
	Men	3.5	−1.3 to 8.3	0.1	−0.6 to 1.0	3.6	−1.1 to 8.4
Diastolic blood pressure, mmHg	HR						
	Pooled	3.2	0.4 to 3.9	3.2	0.4 to 6.0	5.5	2.7 to 8.3
	Women	4.1	−0.2 to 8.3	2.9	1.1 to 5.7	6.9	2.7 to 11.2
	Men	2.0	−1.5 to 5.6	1.7	0.2 to 3.8	3.8	0.1 to 7.4
	rMSSD						
	Pooled	5.4	2.7 to 8.1	0.1	−0.6 to 1.1	5.5	2.7 to 8.3
	Women	5.9	1.8 to 10.0	1.1	−0.1 to 2.8	7.0	2.7 to 11.2
	Men	4.2	0.7 to 7.8	−0.5	−1.5 to 0.4	3.8	0.1 to 7.4
	LFP						
	Pooled	5.4	2.8 to 8.2	0.01	−0.9 to 0.9	5.5	2.7 to 8.3
	Women	5.9	1.8 to 10.0	1.1	−0.3 to 2.8	7.0	2.7 to 11.2
	Men	4.3	0.7 to 7.9	−0.5	−1.7 to 0.3	3.7	0.1 to 7.4
	HFP						
	Pooled	5.3	2.5 to 8.0	0.2	−0.5 to 1.1	5.5	2.7 to 8.3
	Women	5.1	1.0 to 9.2	1.8	0.4 to 3.8	7.0	2.7 to 11.2
	Men	4.3	0.6 to 7.9	−0.5	−1.4 to 0.2	3.7	0.1 to 7.4
	LF/HF						
	Pooled	5.4	2.6 to 8.2	0.1	−0.1 to 0.5	5.5	2.7 to 8.3
	Women	6.4	2.1 to 10.8	0.5	−0.1 to 1.7	7.0	2.7 to 11.2
	Men	3.7	0.04 to 7.4	0.07	−0.5 to 0.8	3.8	0.1 to 7.4

*Note*: Estimated direct, indirect, and total effect between VLBW participants born preterm with VLBW and controls made by mediation analyses where HRV metrics were separately set as mediators (M), group (preterm VLBW vs. term‐controls) as predictor variable (X) and systolic and diastolic blood pressure as outcome variables (Y), adjusted for cohort, age, and sex (if not stratified).

Abbreviations: CI, confidence interval; HFP, high frequency power; HR, mean heart rate; HRV, heart rate variability; LF/HF, ratio between low and high frequency power; LFP, low frequency power; rMSSD, root mean square of successive differences.

### 3.4 Nonparticipant analyses

We have previously published a detailed nonparticipant analysis, which did not show differences in neonatal characteristics between the cohort members who attended this current mid‐adulthood study and those who were invited but did not attend (Jussinniemi et al., 2023). Specifically, for HRV assessment, we now conducted nonparticipant analyses between the participants who were included in the HRV analyses (participants) and those who attended the mid‐adulthood study but were not included in the HRV analyses (nonparticipants; Table S3). We did not find significant differences in neonatal characteristics between the participants and nonparticipants. Preterm born VLBW participants included in the HRV analyses were, however, slightly older at the time of assessment than preterm born VLBW non‐participants (36.5 ± 3.2 vs. 34.9 ± 3.2 years, *p* = 0.01). This age difference was small, and in the absence of other systematic differences may represent a chance finding.

## 4 DISCUSSION

### 4.1 Main findings

We hypothesized that adults born preterm with VLBW have lower cardiac parasympathetic activity than those born at term with normal birth weight and that their higher blood pressure is partly mediated by lower parasympathetic activity. We found that men and women born preterm with VLBW had higher resting heart rates, and women born preterm with VLBW had lower HFP and higher LF/HF as well as systolic and diastolic BP, compared with their term‐born peers with normal birth weight. The results also revealed that, among women, the association between preterm birth with VLBW and higher BP was in part mediated by HRV metrics indicating lower parasympathetic activity.

### 4.2 Interpretations and consistency with previous research

While the altered cardiac autonomic control in preterm infants and children is widely recognized (Bakketeig et al., 1993; Patural et al., 2004), its long‐term persistence has been less clear. HRV has been examined at different ages, and findings suggest a complex and evolving pattern of autonomic regulation. By and large, findings in infants and children born preterm suggest reduced parasympathetic activity (Yiallourou et al., 2013, 2017), although not all findings have been consistent (Haraldsdottir et al., 2018). In young adults in their twenties, similar findings have been seen in those born preterm with extremely low birth weight (birth weight < 920 g, *n* = 60) in Ontario, Canada, compared with 79 control participants (Mathewson et al., 2015). In addition, a similar finding was found among those born in 1986 in Northern Finland including 117 individuals born preterm <34 weeks of gestation, 207 between 34 and 36 weeks and 276 term‐born controls at a mean age of 23 years (Karvonen et al., 2019). However, this was not confirmed in another study of 30 very preterm (VP)/VLBW and 16 full‐term young adults, also in Northern Finland (Björkman et al., 2023). We are unaware of similar studies conducted in adults born preterm approaching middle age; one study in 46‐year‐old in Northern Finland assessed HRV in relation to birth weight and counterintuitively found no association with low birth weight but higher vagal activity among men born post term (Perkiömäki et al., 2016). We are also unaware of any studies formally assessing HRV measures as potential mediators of the association between preterm birth with VLBW and adult cardiometabolic conditions including blood pressure. In the abovementioned study of young adults in Northern Finland (Karvonen et al., 2019) adjustment for HRV measurements weakened the association between preterm birth and BP, which would be consistent with the mediation we found in the present study.

Our findings among women indicate increased sympathetic activity and reduced vagal modulation of heart rate and suggest that this imbalance in autonomic regulation may contribute to elevated blood pressure and potentially by extension an elevated risk of cardiovascular events (Bates et al., 2020; Bigger Jr. et al., 1992; Crump et al., 2019, 2021; Gerritsen et al., 2001). We did not find an association between preterm birth with VLBW and HRV metrics among men. Accordingly, among men, adults born preterm with VLBW did have higher diastolic BP compared with term‐born peers, but this was not mediated by HRV metrics. Existing studies do not provide a clear physiological explanation for the sex differences in heart rate variability. Although with inconsistent results, multiple studies indicate hormonal influences—including sex hormones, ovulatory cycle, menopause, and hormone replacement therapy—might be possible contributors to the sex differences (Smetana & Malik, 2013). Studies also suggest that differences in the autonomic system may stem from variations in neural signaling pathways, receptor activity, neurotransmitters, or reflex responses in different sexes (Moodithaya & Avadhany, 2012). Another meta‐analysis suggested that estrogen, oxytocin, and neural control may provide a basis as underlying mechanisms for sex differences (Koenig & Thayer, 2016).

It is clearly established that being born preterm is a risk factor for elevated blood pressure (Chehade et al., 2018; Crump et al., 2020; Hovi et al., 2016) and for cardiovascular diseases (Lewandowski et al., 2020) later in life. In our main analyses of the total sample, participants born preterm with VLBW had significantly higher systolic (5.3 mmHg) and diastolic (4.5 mmHg) blood pressure. Among women corresponding differences were 6.7 mmHg in systolic BP and 5.1 mmHg in diastolic BP. For context, a 5–10 mmHg increase in diastolic BP is associated with a 34% increased risk for stroke (Law et al., 2003). These results are consistent with previous findings of higher blood pressure in the preterm‐born VLBW population (Hovi et al., 2016) and, as a new finding, suggest that a reduced cardiac parasympathetic function may in part underlie the higher blood pressure at least among VLBW women born preterm.

In general, the function of the cardiac autonomic nervous system and its connection to cardiovascular diseases (CVD) has been extensively studied by using HRV. Although it is well established that people born preterm have higher rates of cardiovascular disease than those born at term (Bates et al., 2020; Bavineni et al., 2019; Chehade et al., 2018; Crump et al., 2021), the underlying mechanisms remain complex and not fully understood. Previous studies have reported a link between impaired cardiac autonomic control and an increased risk of cardiometabolic diseases, morbidity, and mortality (Bigger Jr. et al., 1996; Gerritsen et al., 2001). While autonomic dysfunction may imply a particularly high risk for those who have hypertension (Schroeder et al., 2003; Singh et al., 1998), diabetes (Liao et al., 1995), high cholesterol (Christensen et al., 1999), or a history of CVD (Gerritsen et al., 2001), it also represents a significant risk factor within individuals with no manifest cardiovascular disease (Kiviniemi et al., 2014). Our findings suggest that altered HRV represents one plausible pathway to underlie the increased cardiometabolic risks in adults born preterm.

There have been considerable advancements in HRV measurement techniques. If these techniques allow HRV measurements to be routinely incorporated into clinical practice, this could be particularly important for adults born preterm with VLBW. Healthcare providers treating adults born preterm should remain alert to markers of increased risk for cardiovascular and noncommunicable diseases, such as high blood pressure.

### 4.3 Methodological considerations

To enhance statistical power and precision, the mid‐adulthood assessments were performed in two birth cohorts using standardized methods, with training and audits to ensure consistency in measurements. As a result, our power was appropriate to confirm or exclude moderate or large effects, with adequate power even in sex‐specific analyses. Adults born preterm with VLBW were born in 1978–1988, during the pre‐surfactant era. Our findings are thus most directly relevant for preterm VLBW survivors born at that time, who are now entering middle age. These survivors constitute about one percent of their age group in high resource settings. More contemporary cohorts, born after the introduction of exogenous surfactant and advances in neonatal intensive care, have had improved survival. However, whether and how the improved survival is reflected in childhood and adult cardiovascular function remains poorly known. Moreover, according to our knowledge, no studies have yet examined whether the differences in HRV persist into mid‐adulthood, underscoring the unique contribution of the present follow‐up in this earlier‐born cohort.

In an observational study, we cannot exclude residual confounding. Moreover, we cannot exclude participation bias, although the non‐participant analysis had not detected essential differences in background characteristics of participants and nonparticipants.

## 5 CONCLUSIONS

We conclude that in mid‐adulthood, adults born preterm with VLBW have higher heart rates and blood pressures than those born at term with normal birth weights. Furthermore, women born preterm with VLBW have reduced cardiac parasympathetic activity, which partly mediates their elevated BP. Cardiac autonomic function may constitute one pathway underlying the increased risk of cardiometabolic disease in adults born preterm with VLBW.

## AUTHOR CONTRIBUTIONS

E.K., KA.I.E., and T.S.M. conceived and designed research and performed the experiments; L.J., M.T., and Z.T. analyzed data; E.K., L.J., M.T., and Z.T. interpreted results of experiments; L.J. prepared figures and drafted manuscript; E.K., KA.I.E., L.J., M.T., T.S.M., and Z.T. edited and revised manuscript and approved final version of manuscript.

## FUNDING INFORMATION

The follow‐up study was funded and supported by: Research Council of Finland, 315690, 358384, and 355514 (to E.K.), Alma and K.A Snellman Foundation (to L.J.), European Union's Horizon 2020 Research and Innovation Program: Research on European Children and Adults born Preterm (RECAP Preterm), 733280 (to E.K., KA.I.E., T.S.M.), European Union's Horizon Europe Research and Innovation Program IMPROVE Preterm, 101156325 (to E.K., K.A.I.E., L.J.), Finnish Diabetes Research Foundation (to EK.), Finnish Foundation for Cardiovascular Research (to E.K.), Finnish Medical Foundation (Finska Läkaresällskapet to E.K.), Foundation for Pediatric Research (to E.K.), The Joint Research Committee between St. Olavs hospital and the Faculty of Medicine and Health Sciences, NTNU (FFU, to K.A.I.E, T.S.M.), Mary and Georg C. Ehrnrooth Foundation (to E.K.), Novo Nordisk Foundation (to E.K.), The Research Council of Norway (RCN, Norges Forskningsråd), 33405 (to T.S.M.), Signe and Ane Gyllenberg Foundation (to E.K., L.J.), Sigrid Juselius Foundation (to E.K.), and University of Oulu, University of Oulu Graduate School (to L.J.).

## CONFLICT OF INTEREST STATEMENT

No conflicts of interest are declared by the authors.

## ETHICS STATEMENT

Written informed consent was obtained from all participants who attended the study. The study protocols were approved by the ethics committee at the Helsinki and Uusimaa Hospital district (HUS/1157) and by the Regional Committee for Medical and Health Research Ethics in Central‐Norway (23879). The protocol is registered as ISRCTN77533991.

## Supporting information


Table S1.

Table S2.

Table S3.

## Data Availability

The datasets generated and/or analyzed during the current study include sensitive health data and cannot be made publicly available. Aggregated data are available from the corresponding author upon reasonable request.
