# Supplementary material for: Cardiac autonomic function in adults born preterm with very low birth weight in mid‐adulthood—A two‐country birth cohort study
Source: Physiol Rep. 2025 Oct 29;13(21):e70641. doi: 10.14814/phy2.70641 (PMC12571542; doi:10.14814/phy2.70641)
Supplement: Supplementary file 1 — Table S1. [file PHY2-13-e70641-s001.docx]

Supplemental Table S1. Sensitivity analysis of mean differences in HRV measurement between adults born preterm with VLBW and control participants.

|  | **Group** | **VLBW/**  **Term-controls** | **VLBW** | **Term-control (reference)** |  |
| --- | --- | --- | --- | --- | --- |
|  |  | n | Mean (SD) | Mean (SD) | Mean difference  (95% Confidence interval^b^) |
| Resting HR (bpm) |  |  |  |  |  |
| Pooled | 0 | 107/142 | 73.7 (10.5) | 67.5 (10.0) | **5.9 (3.4 to 8.4)** |
| women | 0 | 59/81 | 75.8 (8.7) | 69.4 (10.4) | **6.6 (3.2 to 9.9)** |
| men | 0 | 48/61 | 71.1 (11.9) | 65.0 (8.9) | **5.3 (1.5 to 9.1)** |
|  |  |  |  |  |  |
| Pooled | 1 | 70/123 | 71.5 (10.8) | 67.2 (9.5) | **4.4 (1.6 to 7.2)** |
| women | 1 | 35/74 | 74.7 (8.9) | 69.3 (9.8) | **5.6 (1.7 to 9.6)** |
| men | 1 | 35/49 | 68.2 (10.5) | 64.0 (8.0) | 2.7 (-1.1 to 6.5) |
|  |  |  |  |  |  |
| pooled | 2 | 77/124 | 72.3 (10.9) | 67.2 (9.4) | 5.3 (2.5 to 8.1) |
| women | 2 | 41/75 | 75.0 (9.0) | 69.3 (9.7) | **6.0 (2.3 to 9.7)** |
| men | 2 | 36/49 | 69.2 (12.0) | 64.0 (8.0) | 4.2 (-0.30 to 8.5) |
|  |  |  |  |  |  |
| pooled | 3 | 92/134 | 73.3 (10.5) | 67. 4 (9.9) | **5.6 (3.0 to 8.2)** |
| women | 3 | 52/79 | 76.2 (8.6) | 69.7 (10.4) | **6.5 (3.02 to 10.0)** |
| men | 3 | 40/55 | 69.6 (64.1) | 64.1 (8.3) | **4.4 (0.56 to 8.26)** |
|  |  |  |  |  |  |
| Pooled | 4 | 100/141 | 73.1 (10.2) | 67.6 (10.0) | **5.1 (2.6 to 7.6)** |
| women | 4 | 55/80 | 75.6 (8.8) | 65.6 (10.4) | **6.1 (2.7 to 9.6)** |
| men | 4 | 45/61 | 69.9 (11.1) | 65.0 (8.9) | **4.2 (0.49 to 7.82)** |
| rMSSD (ms)^a^ |  |  |  |  |  |
| Pooled | 0 | 107/142 | 44.7 (2.3) | 47.5 (2.0) | -3.0% (-18.9% to 17.4%) |
| women | 0 | 59/81 | 37.0 (1.9) | 46.1 (2.1) | -18.2% (-35.6% to 4.1%) |
| men | 0 | 48/61 | 55.7 (2.6) | 49.4 (1.9) | 18.5% (-10.4% to 58.4%) |
|  |  |  |  |  |  |
| pooled | 1 | 70/123 | 3.85 (0.81) | 3.89 (0.68) | -0.01% (-0.23% to 0.21%) |
| women | 1 | 35/74 | 3.66 (0.64) | 3.86 (0.72) | -0.19% (-0.48% to 0.10%) |
| men | 1 | 35/49 | 4.03 (0.91) | 3.93 (0.62) | 0.22% (-0.10% to 0.54%) |
|  |  |  |  |  |  |
| pooled | 2 | 77/124 | 3.83 (0.81) | 3.88 (0.68) | -0.02% (-0.23% to 0.19%) |
| women | 2 | 41/75 | 3.64 (0.66) | 3.85 (0.72) | -0.20% (-0.48% to 0.07%) |
| men | 2 | 36/49 | 4.06 (0.90) | 3.93 (0.62) | 0.23% (-0.08% to 0.54%) |
|  |  |  |  |  |  |
| pooled | 3 | 92/134 | 3.76 (0.78) | 3.87 (0.69) | -0.07% (-0.26% to 0.13%) |
| women | 3 | 52/79 | 3.58 (0.65) | 3.83 (0.74) | -0.22% (-0.48% to 0.03%) |
| men | 3 | 40/55 | 3.98 (0.89) | 3.92 (0.60) | 0.15% (-0.15% to 0.44%) |
|  |  |  |  |  |  |
| pooled | 4 | 100/141 | 3.82 (0.83) | 3.86 (0.68) | 0.003% (-0.19% to 0.19%) |
| women | 4 | 55/80 | 3.60 (0.60) | 3.83 (0.73) | -0.20% (-0.44% to 0.05%) |
| men | 4 | 45/61 | 4.07 (0.97) | 3.90 (0.61) | 0.23% (-0.6% to 0.52%) |
| Low frequency power (ms^2^)^a^ |  |  |  |  |  |
| pooled | 0 | 107/142 | 7.08 (1.30) | 7.11 (1.06) | 0.001% (-0.29% to 0.29%) |
| women | 0 | 59/81 | 6.73 (0.99) | 6.99 (1.07) | -0.27% (-0.62% to 0.10%) |
| men | 0 | 48/61 | 7.51 (1.52) | 7.27 (1.04) | 0.30% (-0.18% to 0.79%) |
|  |  |  |  |  |  |
| pooled | 1 | 70/123 | 7.22 (1.25) | 7.19 (1.02) | 0.009% (-0.32% to 0.33%) |
| women | 1 | 35/74 | 6.78 (0.93) | 7.01 (1.03) | -0.30% (-0.12% to 0.90%) |
| men | 1 | 35/49 | 7.66 (1.38) | 7.39 (0.97) | 0.39% (-0.12% to 0.90%) |
|  |  |  |  |  |  |
| pooled | 2 | 77/124 | 7.15 (1.28) | 7.18 (1.02) | -0.04% (-0.36% to 0.27%) |
| women | 2 | 41/75 | 6.70 (1.02) | 7.05 (1.03) | 6.1% (1.60% to 10.7%) |
| men | 2 | 36/49 | 7.65 (1.36) | 7.39 (0.97) | 0.36% (-0.14% to 0.86%) |
|  |  |  |  |  |  |
| pooled | 3 | 92/134 | 7.06 (1.23) | 7.15 (1.04) | -0.07% (-0.37% to 0.22%) |
| women | 3 | 52/79 | 6.71 (1.00) | 7.00 (1.08) | -0.28% (-0.66% to 0.10%) |
| men | 3 | 40/55 | 7.50 (1.37) | 7.37 (0.94) | 0.21% (-0.26% to 0.67%) |
|  |  |  |  |  |  |
| pooled | 4 | 100/141 | 7.14 (1.31) | 7.12 (1.07) | 0.06% (-0.24% to 0.36%) |
| women | 4 | 55/80 | 6.74 (0.99) | 7.00 (1.08) | -0.25% (-0.62% to 0.12%) |
| men | 4 | 45/61 | 7.62 (1.48) | 7.27 (1.04) | 0.41% (-0.07% to 0.89%) |
| High frequency power (ms^2^)^a^ |  |  |  |  |  |
| pooled | 0 | 107/142 | 6.66 (1.67) | 6.81 (1.28) | -0.10% (-0.47% to 0.27%) |
| women | 0 | 59/81 | 6.28 (1.28) | 6.84 (1.30) | **-0.57% (-1.02% to -0.12%)** |
| men | 0 | 48/61 | 7.13 (1.97) | 6.78 (1.26) | -0.46% (-1.05% to 0.14%) |
|  |  |  |  |  |  |
| pooled | 1 | 70/123 | 6.77 (1.61) | 6.88 (1.28) | -0.07% (-0.49% to 0.36%) |
| women | 1 | 35/74 | 6.38 (1.28) | 6.89 (1.29) | -0.52% (-1.06% to 0.01%) |
| men | 1 | 35/49 | 7.15 (1.83) | 6.86 (1.27) | 0.50% (-0.16 %to 1.16%) |
|  |  |  |  |  |  |
| pooled | 2 | 77/124 | 6.71 (1.62) | 6.87 (1.28) | -0.12% (-0.53% to 0.29%) |
| women | 2 | 41/75 | 6.30 (1.29) | 6.88 (1.29) | **-0.59% (-1.10% to -0.09%)** |
| men | 2 | 36/49 | 7.18 (1.82) | 6.86 (1.27) | 0.51% (-0.14% to 1.16%) |
|  |  |  |  |  |  |
| pooled | 3 | 92/134 | 6.59 (1.56) | 6.84 (1.28) | -0.19% (-0.57% to 0.19%) |
| women | 3 | 52/79 | 6.24 (1.27) | 6.84 (1.32) | **-0.60% (-1.06% to -0.13%)** |
| men | 3 | 40/55 | 7.04 (1.80) | 6.84 (1.24) | 0.36% (-0.25% to 0.96%) |
|  |  |  |  |  |  |
| pooled | 4 | 100/141 | 6.71 (1.67) | 6.81 (1.28) | -0.03% (-0.41% to 0.34%) |
| women | 4 | 55/80 | 6.28 (1.24) | 6.83 (1.31) | **-0.56% (-1.01% to -0.10%)** |
| men | 4 | 45/61 | 7.24 (1.97) | 6.78 (1.26) | 0.57% (-0.03% to 1.17%) |
| LF/HF ^a^ |  |  |  |  |  |
| pooled | 0 | 107/142 | 0.42 (0.73) | 0.30 (0.76) | 0.10% (-0.09% to 0.29%) |
| women | 0 | 59/81 | 0.45 (0.68) | 0.16 (0.82) | **0.31% (0.04% to 0.57%)** |
| men | 0 | 48/61 | 0.38 (0.80) | 0.49 (0.64) | -0.15% (-0.42% to 0.12%) |
|  |  |  |  |  |  |
| pooled | 1 | 70/123 | 0.45 (0.72) | 0.32 (0.79) | 0.08% (-0.15% to 0.03%) |
| women | 1 | 35/74 | 0.39 (0.71) | 0.18 (0.84) | 0.22% (-0.11% to 0.56%) |
| men | 1 | 35/49 | 0.51 (0.75) | 0.53 (0.66) | -0.12% (-0.42% to 0.18%) |
|  |  |  |  |  |  |
| pooled | 2 | 77/124 | 0.44 (0.71) | 0.31 (0.79) | 0.08% (-0.14% to 0.30%) |
| women | 2 | 41/75 | 0.41 (0.67) | 0.17 (0.83) | 0.24% (-0.07% to 0.55%) |
| men | 2 | 36/49 | 0.47 (0.77) | 0.53 (0.66) | -0.15% (-0.45% to 0.15%) |
|  |  |  |  |  |  |
| pooled | 3 | 92/134 | 0.46 (0.72) | 0.31 (0.77) | 0.12% (-0.08% to 0.32%) |
| women | 3 | 52/79 | 0.47 (0.66) | 0.16 (0.81) | **0.31% (0.04% to 0.59%)** |
| men | 3 | 40/55 | 0.46 (0.78) | 0.53 (0.64) | -0.15% (-0.43% to 0.14%) |
|  |  |  |  |  |  |
| pooled | 4 | 100/141 | 0.43 (0.72) | 0.31 (0.76) | 0.09% (-0.10% to 0.29%) |
| women | 4 | 55/80 | 0.47 (0.65) | 0.17 (0.81) | **0.31% (0.04% to 0.58%)** |
| men | 4 | 45/61 | 0.38 (0.79) | 0.49 (0.64) | -0.16% (-0.43% to 0.11%) |
| Systolic blood pressure (mmHg) |  |  |  |  |  |
| pooled | 0 | 105/141 | 117.4 (14.8) | 111.5 (12.2) | **5.3 (2.2 to 8.4)** |
| women | 0 | 57/80 | 113.3 (14.5) | 106.00 (10.1) | **6.7 (2.5 to 10.9)** |
| men | 0 | 48/61 | 122.3 (13.9) | 118.8 (10.8) | 3.6 (-1.14 to 8.4) |
|  |  |  |  |  |  |
| pooled | 1 | 70/122 | 117.1 (14.9) | 111.4 (12.1) | **4.1 (0.50 to 7.61)** |
| women | 1 | 35/73 | 113.5 (14.1) | 106.0 (10.0) | **6.9 (2.18 to 11.52)** |
| men | 1 | 45/49 | 120.7 (14.1) | 119.5 (10.4) | 1.1 (-4.4 to 6.5) |
|  |  |  |  |  |  |
| pooled | 2 | 76/123 | 116.7 (14.8) | 111.3 (12.1) | **4.0 (0.57 to 7.49)** |
| women | 2 | 40/74 | 112.5 (14.0) | 105.9 (9.9) | **5.9 (1.50 to 10.3)** |
| men | 2 | 36/49 | 121.4 (14.5) | 119.5 (10.4) | 1.9 (-3.54 to 7.43) |
|  |  |  |  |  |  |
| pooled | 3 | 91/133 | 116.8 (15.2) | 111.5 (11.9) | **4.6 (1.32 to 7.90)** |
| women | 3 | 51/78 | 112.7 (14.6) | 106.2 (9.9) | **5.8 (1.58 to 10.1)** |
| men | 3 | 40/55 | 122.0 (14.5) | 118.9 (10.5) | 3.1 (-2.04 to 8.32) |
|  |  |  |  |  |  |
| pooled | 4 | 99/140 | 117.0 (15.1) | 111.7 (12.0) | **4.8 (1.57 to 7.94)** |
| women | 4 | 54/79 | 112.7 (14.5) | 106. 3 (9.9) | 5.7 (1.55 to 9.94) |
| men | 4 | 45/61 | 122.2 (14.3) | 118.8 (10.8) | 3.5 (-1.38 to 8.47) |
| Diastolic blood pressure (mmHg) |  |  |  |  |  |
| pooled | 0 | 105/141 | 80.8 (11.2) | 75.8 (8.3) | **4.5 (2.0 to 7.0)** |
| women | 0 | 57/80 | 79.8 (11.3) | 74.4 (8.5) | **5.1 (1.7 to 8.5)** |
| men | 0 | 48/61 | 81.9 (11.2) | 77.8 (7.7) | **3.8 (0.15 to 7.4)** |
|  |  |  |  |  |  |
| pooled | 1 | 70/122 | 79.8 (10.6) | 75.7 (8.4) | **3.2 (0.42 to 5.88)** |
| women | 1 | 35/73 | 79.2 (10.9) | 74.1 (8.3) | **4.5 (0.78 to 8.33)** |
| men | 1 | 45/49 | 80.5 (10.5) | 78.1 (7.9) | 1.5 (-2.5 to 5.5) |
|  |  |  |  |  |  |
| pooled | 2 | 76/123 | 80.0 (10.8) | 75.7 (8.3) | **3.5 (0.88 to 6.2)** |
| women | 2 | 40/74 | 79.2 (10.8) | 74.1 (8.3) | **4.6 (0.97 to 8.15)** |
| men | 2 | 36/49 | 81.0 (10.8) | 78.1 (7.9) | 2.3 (-1.80 to 6.39) |
|  |  |  |  |  |  |
| pooled | 3 | 91/133 | 80.4 (11.5) | 75.8 (8.3) | **4.1 (1.48 to 6.70)** |
| women | 3 | 51/78 | 79.4 (11.3) | 74.4 (8.5) | **4.5 (1.01 to 8.00)** |
| men | 3 | 40/55 | 81.2 (11.9) | 77.8 (7.7) | 3.6 (-0.46 to 7.62) |
|  |  |  |  |  |  |
| pooled | 4 | 99/140 | 80.5 (11.4) | 75.9 (8.3) | **4.1 (1.60 to 6.61)** |
| women | 4 | 54/79 | 79.5 (11.3) | 74.5 (8.5) | **4.55 (1.09 to 8.00)** |
| men | 4 | 45/61 | 81.7 (11.5) | 77.8 (7.7) | 3.6 (-0.18 to 7.29) |

Means and mean difference comparisons between adults born preterm with VLBW participants and term-born controls made by linear regressions adjusted for age, cohort and sex (if not stratified). Mean differences for rMSSD, low frequency power, high frequency power and LF/HF have been calculated from log transformed values, back-transformed and expressed as percentage difference. Explanations for different groups : 0: Group without exclusions (participants with the HRV data), sensitivity analysis group 1: exclusion of participants with cp, type II diabetes mellitus, heart disease, users of ATC=C09, Inhaled adrenergic bronchodilators (ATC=R03AC), B-blockers (ATC=C07A), type II diabetes mellitus medication (ATC=A10B), anti-cholesterol medicine (ATC=C10), psycholeptics (ATC=N05), psychostimulants (ATC=N06B), sensitivity analysis group 2: exclusion of participants with cp, type II diabetes mellitus, heart disease, users of ATC=C09, inhaled adrenergic bronchodilators (ATC=R03AC), B -blockers (ATC=C07A), type II diabetes mellitus medication (ATC=A10B) and anti-cholesterol medicine (ATC=C10), sensitivity analysis group 3: exclusion of participants with cp, heart disease, users of ATC=C09, B-blockers (ATC=C07A), sensitivity analysis model 4: exclusion of participants with CP. Abbreviations: bpm: beats per minute, HFP: high frequency power, HR: mean heart rate, LFP: Low frequency power, LF/HF: ratio between low and high frequency power, ms: millisecond, ms^2^: square millisecond, SD: standard deviation, rMSSD: root mean square of successive differences,  VLBW: very low birth weight.

^a^Means (SD) for rMSSD, LFP, HFP and LF/HF are geometric means.

^b^Mean differences for rMSSD, LFP, HFP and LF/HF have been calculated from log transformed values, back-transformed and expressed as percentage difference.
