# Supplementary material for: Cardiac autonomic function in adults born preterm with very low birth weight in mid‐adulthood—A two‐country birth cohort study
Source: Physiol Rep. 2025 Oct 29;13(21):e70641. doi: 10.14814/phy2.70641 (PMC12571542; doi:10.14814/phy2.70641)
Supplement: Supplementary file 2 — Table S2. [file PHY2-13-e70641-s003.docx]

Supplemental Table S2. Mean differences in HRV measurements between adults born preterm with VLBW and control participants with additional adjustment models.

|  |  | **VLBW/**  **term** | **VLBW** | **Term (reference)** |  |
| --- | --- | --- | --- | --- | --- |
|  | Model | n | Mean (SD) | Mean (SD) | Mean difference (95% Confidence interval^b^) |
| Resting HR (bpm) |  |  |  |  |  |
| Pooled | 1 | 107/142 | 73.7 (10.5) | 67.5 (10.0) | **5.9 (3.4 to 8.4)** |
| women | 1 | 59/81 | 75.8 (8.7) | 69.4 (10.4) | **6.6 (3.2 to 9.9)** |
| men | 1 | 48/61 | 71.1 (11.9) | 65.0 (8.9) | **5.3 (1.5 to 9.1)** |
| Pooled | 2 |  |  |  | **5.6 (3.0 to 8.2)** |
| women | 2 |  |  |  | **6.3 (2.8 to 9.7)** |
| men | 2 |  |  |  | **5.1 (1.1 to 9.1)** |
| women | 3 | 59/81 | 75.8 (8.7) | 69.4 (10.4) | **6.6 (3.2 to 9.9)** |
| rMSSD (ms)^a^ |  |  |  |  |  |
| pooled | 1 | 107/142 | 3.80 (0.83) | 3.86 (0.68) | -0.03% (-0.21% to 0.16%) |
| women | 1 | 59/81 | 3.61 (0.65) | 3.83 (0.73) | -0.20% (-0.44% to 0.04%) |
| men | 1 | 48/61 | 4.02 (0.97) | 3.90 (0.62) | 0.17% (-0.11% to 0.46%) |
| pooled | 2 |  |  |  | -0.005% (-0.20% to 0.19%) |
| women | 2 |  |  |  | -0.17% (-0.41% to 0.08%) |
| men | 2 |  |  |  | 0.18% (-0.14% to 0.49%) |
| women | 3 | 59/81 | 3.61 (0.65) | 3.83 (0.73) | -0.21% (-0.45% to 0.03%) |
| Low frequency power (ms^2^)^a^ |  |  |  |  |  |
| pooled | 1 | 107/142 | 7.08 (1.30) | 7.11 (1.06) | 0.001% (-0.29% to 0.29%) |
| women | 1 | 59/81 | 6.73 (0.99) | 6.99 (1.07) | -0.27% (-0.62% to 0.10%) |
| men | 1 | 48/61 | 7.51 (1.52) | 7.27 (1.04) | 0.30% (-0.18% to 0.79%) |
| pooled | 2 |  |  |  | 0.03% (-0.28% to 0.34%) |
| women | 2 |  |  |  | -0.23% (-0.61% to 0.14%) |
| men | 2 |  |  |  | 0.33 (-0.20 to 0.85) |
| women | 3 | 59/81 | 6.73 (0.99) | 6.99 (1.07) | -0.30 (-0.65 to 0.06) |
| High frequency power (ms^2^)^a^ |  |  |  |  |  |
| pooled | 1 | 107/142 | 6.66 (1.67) | 6.81 (1.28) | -0.10% (-0.47% to 0.27%) |
| women | 1 | 59/81 | 6.28 (1.28) | 6.84 (1.30) | **-0.57% (-1.02% to -0.12%)** |
| men | 1 | 48/61 | 7.13 (1.97) | 6.78 (1.26) | -0.46% (-1.05% to 0.14%) |
| pooled | 2 |  |  |  | -0.07% (-0.45% to 0.38%) |
| women | 2 |  |  |  | **-0.51% (-0.96% to -0.05%)** |
| men | 2 |  |  |  | 0.44% (-0.20% to 1.09%) |
| women | 3 | 59/81 | 6.28 (1.28) | 6.84 (1.30) | **-0.60% (-1.05% to -0.15%)** |
| LF/HF^a^ |  |  |  |  |  |
| pooled | 1 |  | 0.42 (0.73) | 0.30 (0.76) | 0.10% (-0.09% to 0.29%) |
| women | 1 |  | 0.45 (0.68) | 0.16 (0.82) | **0.31% (0.04% to 0.57%)** |
| men | 1 |  | 0.38 (0.80) | 0.49 (0.64) | -0.15% (-0.42% to 0.12%) |
| pooled | 2 |  |  |  | 0.10% (-0.10% to 0.29%) |
| women | 2 |  |  |  | **0.27% (0.008% to 0.54%)** |
| men | 2 |  |  |  | -0.12% (-0.40% to 0.17%) |
| women | 3 | 59/81 | 0.45 (0.68) | 0.16 (0.82) | **0.30% (0.03% to 0.57%)** |
| Systolic blood pressure (mmHg) |  |  |  |  |  |
| pooled | 1 | 105/141 | 117.4 (14.8) | 111.5 (12.2) | **5.3 (2.2 to 8.4)** |
| women | 1 | 57/80 | 113.3 (14.5) | 106.00 (10.1) | **6.7 (2.5 to 10.9)** |
| men | 1 | 48/61 | 122.3 (13.9) | 118.8 (10.8) | 3.6 (-1.14 to 8.4) |
| pooled | 2 |  |  |  | **5.2 (1.7 to 8.8)** |
| women | 2 |  |  |  | **5.8 (1.6 to 10.1)** |
| men | 2 |  |  |  | 3.3 (-1.7 to 8.2) |
| women | 3 | 57/80 | 113.3 (14.5) | 106.00 (10.1) | **7.0 (2.9 to 11.2)** |
| Diastolic blood pressure (mmHg) |  |  |  |  |  |
| pooled | 1 | 105/141 | 80.8 (11.2) | 75.8 (8.3) | **4.5 (2.0 to 7.0)** |
| women | 1 | 57/80 | 79.8 (11.3) | 74.4 (8.5) | **5.1 (1.7 to 8.5)** |
| men | 1 | 48/61 | 81.9 (11.2) | 77.8 (7.7) | **3.8 (0.15 to 7.4)** |
| pooled | 2 |  |  |  | **4.0 (1.5 to 6.6)** |
| women | 2 |  |  |  | **4.4 (0.9 to 7.9)** |
| men | 2 |  |  |  | 3.2 (-0.6 to 7.0) |
| women | 3 | 59/81 | 79.8 (11.3) | 74.4 (8.5) | **5.4 (2.0 to 8.7)** |

Mean difference comparisons between adults born preterm with VLBW participants and term-born controls are made by linear regression with different adjustment models; model 1: adjusted for cohort, age and sex (if not stratified), model 2: adjusted for cohort, age, sex (if not stratified) and parental education, model 3: adjusted for cohort, age, sex (if not stratified) and hormonal contraception. Mean differences for rMSSD, low frequency power, high frequency power and LF/HF have been calculated from log transformed values, back-transformed and expressed as percentage difference with same adjustment model. Abbreviations: bpm: beats per minute, HFP: high frequency power; HR: mean heart rate, LFP: Low frequency power, LF/HF: ratio between low and high frequency power, ms: millisecond, ms^2^: square millisecond, SD: standard deviation, rMSSD: root mean square of successive differences, VLBW: very low birth weight.

^a^Means (SD) for rMSSD, LFP, HFP and LF/HF are geometric means.

^b^Mean differences for rMSSD, LFP, HFP and LF/HF have been calculated from log transformed values, back-transformed and expressed as percentage difference.
