# Supplementary material for: Cardiac autonomic function in adults born preterm with very low birth weight in mid‐adulthood—A two‐country birth cohort study
Source: Physiol Rep. 2025 Oct 29;13(21):e70641. doi: 10.14814/phy2.70641 (PMC12571542; doi:10.14814/phy2.70641)
Supplement: Supplementary file 3 — Table S3. [file PHY2-13-e70641-s002.docx]

Supplemental Table S3. Non-participant analyses between the participants who were included in the HRV analysis and those who attended the mid-adulthood assessment but were not included in the HRV analysis.

|  | **Included in the HRV analysis**  **“participant”** | **Attended mid-adulthood assessment but not HRV**  **“non-participant”** |  |
| --- | --- | --- | --- |
| VLBW, n | 107 | 30 |  |
| Control, n | 142 | 16 |  |
| Maternal characteristics | **Mean (SD) or n (%)** | **Mean (SD) or n (%)** | **p-value** |
| Age of the mother (years) |  |  |  |
| VLBW | 29.8 (4.8) | 29.3 (4.5) | 0.57 |
| Control | 30.2 (5.0) | 29.7 (2.8) | 0.58 |
| Smoking during pregnancy^a^ |  |  |  |
| VLBW | 13 (23.6%) | 1 (9.1%) | 0.28 |
| Control | 11 (17.2%) | 0 (0%) | 0.27 |
| Parental education |  |  |  |
| VLBW |  |  | 0.005 |
| Control |  |  | 0.41 |
| Basic or less |  |  |  |
| VLBW | 17 (16.3%) | 13 (44.8%) |  |
| Control | 15 (11.5%) | 3 (20.0%) |  |
| Upper secondary |  |  |  |
| VLBW | 20 (19.2%) | 3 (10.3%) |  |
| Control | 28 (21.4%) | 2 (13.3%) |  |
| Lower-lever tertiary |  |  |  |
| VLBW | 38 (36.5%) | 4 (13.8%) |  |
| Control | 35 (26.7%) | 6 (40.0%) |  |
| Upper-level tertiary |  |  |  |
| VLBW | 29 (27.9%) | 9 (31%) |  |
| Control | 53 (40.5%) | 4 (26.7%) |  |
| **Study participant background** |  |  |  |
| Sex, women |  |  |  |
| VLBW | 59 (55.1%) | 19 (63.3%) | 0.42 |
| Control | 81 (57.0%) | 12 (75.0%) | 0.17 |
| Gestational age (weeks) |  |  |  |
| VLBW | 29.34 (2.5) | 29.09 (2.8) | 0.63 |
| Control | 40 (1.1) | 40.31 (1.4) | 0.31 |
| Birth weight (g) |  |  |  |
| VLBW | 1158 (220) | 1112 (240) | 0.32 |
| Control | 3646 (464) | 3684 (592) | 0.77 |
| Birth weight SD score Finnish reference |  |  |  |
| VLBW | -1.3 (1.6) | -1.4 (1.7) | 0.74 |
| Control | 0.1 (1.0) | 0.2 (1.1) |  |
| Birth weight SD score Norwegian reference |  |  |  |
| VLBW | -1.1 (1.1) | -1.1 (1.2) | 0.80 |
| Control | 0.04 (1.0) | 0.1 (1.1) | 0.75 |
| Cerebral palsy |  |  |  |
| VLBW | 7 (6.5%) | 3 (10%) | 0.52 |
| Control | - | - | - |
| Ventilator treatment (days) |  |  |  |
| VLBW | 8.6 (14.9) | 10.2 (19.0) | 0.64 |
| Control | - | - | - |
| Supplemental oxygen (days) |  |  |  |
| VLBW | 19.7 (25.0) | 39.3 (75.2) | 0.19 |
| Control | - | - | - |
| Age at discharge from hospital (days) |  | - |  |
| VLBW | 67.7 (25.5) | 84.3 (69.1) | 0.25 |
| Control | - | - | - |
| Bronchopulmonary dysplasia |  |  |  |
| Defined as supplemental oxygen > 28 days |  |  |  |
| VLBW | 30 (29.4%) | 11 (40.7%) | 0.26 |
| Control | - | - | - |
| Defined as supplemental oxygen > 36 weeks |  |  |  |
| VLBW | 8 (7.8%) | 5 (18.5%) | 0.10 |
| Control | - | - | - |
| Diagnosed by a clinician |  |  |  |
| VLBW | 19 (24.1%) | 8 (44.4%) | 0.081 |
| Control | - | - | - |
| **Study participant current characteristics** |  |  |  |
| Age (years) |  |  |  |
| VLBW | 36.5 (3.2) | 34.9 (3.2) | 0.01 |
| Control | 35.8 (3.3) | 34.9 (2.6) | 0.31 |
| Educational attainment |  |  |  |
| VLBW |  |  | 1.00 |
| Control |  |  | 0.03 |
| Lower (ISCED levels 1-2) |  |  |  |
| VLBW | 4 (3.7%) | 1 (3.3%) |  |
| Control | 2 (1.4%) | 2 (12.5%) |  |
| Intermediate (ISCED levels 3-5) |  |  |  |
| VLBW | 51 (47.7%) | 14 (46.7%) |  |
| Control | 47 (33.1%) | 2 (12.5%) |  |
| Lower tertiary or higher (ISCED levels 6-8) |  |  |  |
| VLBW | 52 (48.6%) | 15 (50%) |  |
| Control | 93 (65.5%) | 12 (75%) |  |
| Height (cm) |  |  |  |
| VLBW | 168.3 (9.8) | 165.8 (10.0) | 0.22 |
| Control | 173.8 (9.6) | 171.6 (10.5) | 0.43 |
| BMI kg/m^2^ |  |  |  |
| VLBW | 26.2 (6.2) | 28.1 (8.1) | 0.16 |
| Control | 25.6 (4.4) | 23.8 (3.2) | 0.12 |
| Smoking daily (yes/no) |  |  |  |
| VLBW | 12 | 3 |  |
| Control | 15 | 1 |  |
| Currently pregnant (women) |  |  |  |
| VLBW | - | 4 (14.8%) |  |
| Control | - | 2 (13.3%) |  |
| Hormonal contraception |  |  |  |
| VLBW | 15 (30%) | 5 (29.4%) | 0.57 |
| Control | 29 (39.7%) | 6 (50%) | 0.66 |

Values are means (SD), comparisons between participants and non-participants are made by x^2^ and t-tests. Abbreviations: BMI: body mass index, HeSVA: Helsinki Study of Very Low Birth weight Adults, ISCED: International Standard Classification of Education, NTNU: NTNU Low Birth Weight in a Lifetime Perspective Study, SD: standard deviation, VLBW: very low birth weight.

^a^Data available only from HeSVA participants.
